# Influence of Machine Anvil Wear on Charpy Test Results

**DOI:** 10.6028/jres.125.015

**Published:** 2020-12-05

**Authors:** Enrico Lucon, Raymond L. Santoyo

**Affiliations:** 1 National Institute of Standards and Technology, Boulder, CO 80305, USA

**Keywords:** absorbed energy, anvil radius, ASTM E23, Charpy, ISO 148, machine anvils, span, wear.

## 1 Summary

We investigated the influence of the state of wear of Charpy machine anvils on test results by performing impact tests on NIST specimens of three energy levels with a machine equipped with new anvils (compliant with both ASTM E23 [1] and ISO 148-2 [2]) and worn anvils (anvil corner radii and distance outside ASTM tolerances, but within ISO tolerances).

The results obtained, statistically analyzed, unequivocally show that worn anvils tend to increase absorbed energy at all energy levels. On the other hand, data variability does not appear to be significantly affected by anvil wear.

This study represents NIST contribution to an international effort spearheaded by the Japan Iron and Steel Federation Standardization Center (Tokyo, Japan).

## 2. Data Specifications

**Table:** 

**NIST Operating Unit(s)**	Material Measurement Laboratory (MML), Div. 647
**Format**	CSV files
**Instrument**	Large-capacity impact machine in the NIST Charpy Laboratory in Boulder, CO. Machine characteristics: potential energy (capacity) 953 J, impact speed 5.47 m/s, striker with 8 mm striking edge (not instrumented).
**Spatial or Temporal Elements**	N/A
**Data Dictionary**	N/A
**Accessibility**	All datasets submitted to Journal of Research of NIST are publicly available.
**License**	https://www.nist.gov/director/licensing

## 3 Methods

Three types of NIST Charpy specimens were selected for this study:

• low-energy specimens (4340 steel, lot LL-157),
  • high-energy specimens (4340 steel, lot HH-170), and
  • super-high-energy specimens (9310 steel, lot SH-56).

Tests were performed at room temperature (21 °C ± 1 °C) on a Charpy machine with a capacity (potential energy) of 953.6 J and an impact speed of 5.47 m/s. The machine was equipped with a non-instrumented striker with 8 mm radius of the striking edge.

For each anvil condition (new and worn), 60 Charpy tests were conducted (25 at low- and high-energy level, 10 at super-high-energy level).

Four pairs of used (worn) anvils were available for the chosen machine. In order to select the best pair for investigating the “worn” condition, we obtained replicas (molds) of the used anvil profiles by means of a silicone-type material, generally used for dental impressions. Once dried and solidified, replicas were sectioned in two positions (P1, P2), both inside the contact area between anvil and specimen (worn area).

**Fig. 1:**
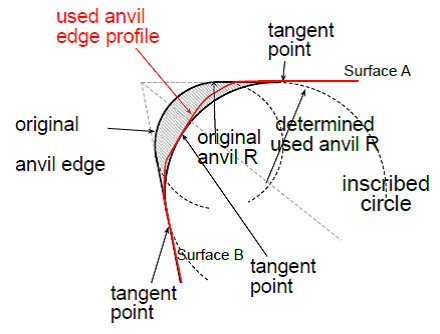
Measurement of worn anvil radius.

In accordance with Fig. 1, taken from the Data Collection Protocol, radius measurements (*r*_P1_ and *r*_P2_ for the two replica sections) were obtained by taking pictures of the replica sections and inscribing a circle on each picture, tangent to both sides of the anvil. The average of the radii of the inscribed circles for the two replica sections was taken as the radius for each worn anvil.

Based on the measurements performed, anvils with average radii $\overset{-}{r}$ = 1.38 mm and 1.33 mm were selected for the tests in the worn condition. They represent a case that is clearly beyond the ASTM allowable limit (*r*_max_ *=* 1.05 mm), but well within the ISO limit (*r*_max_ = 1.50 mm). Moreover, the difference between the measured radii and the two limits is large enough to provide some margin with respect to possible measurement uncertainties.

After the worn anvils were installed on the Charpy machine, we used a caliper to measure their distance (span), which was found to be equal to 40.10 mm. This is invalid according to ASTM E23 (> 40.05 mm), but acceptable per ISO 148-2 (≤ 40.20 mm).

Three datasets in CSV format have been made available, one for each tested energy level (low, high, and super-high energy). At each energy level, the files contain Charpy results (absorbed energy, *KV*, in J) obtained with worn [column A] and new [column B] anvils. In columns D-H, the results of the following statistical analyses [3,4] are provided:

• Rows 1-12: Two-sample *F*-test for the equality of variances.
• Rows 14-29: Two-sample *t*-test for the equality of means (assuming equal variances).

The analyses performed and the results obtained are presented in detail in NIST Technical Note 2089 [5].

## 4 Impact

Our test results at different energy levels showed that worn anvils (outside ASTM tolerances but well within ISO tolerances, in terms of both corner radii and distance) tend to increase absorbed energy in a statistically significant manner. The statistical analyses consisted in a two-sample *t*-test to assess whether the influence of anvil condition on Charpy absorbed energy values was statistically significant.

These results do not support the proposal to relax the ASTM E23 upper tolerances on anvil radius and span to match the ISO 148-2 values (1.50 mm and 10.20 mm, respectively). On the contrary, should test results from other laboratories confirm our conclusions, it would be appropriate to propose a revision of ISO 148-2 aimed at tightening these upper tolerances.

## References

[1] ASTM International (2018) ASTM E23-18 - Standard Test Methods for Notched Bar Impact Testing of Metallic Materials (ASTM International, West Conshohocken, PA).

[2] International Organization for Standardization (2016) ISO 148-2:2016 - Metallic materials - Charpy pendulum impact test - Part 2: Verification of testing machines (International Standardization Organization, Geneva, Switzerland).

[3] Snedecor GW, Cochran WG (1989) Statistical method (Iowa State University Press), 8th Ed.

[4] NIST/SEMATECH e-Handbook of Statistical Methods, https://www.itl.nist.gov/div898/handbook/, update April 2012.

[5] Lucon E, Santoyo RL (2020), Influence of Anvil Wear on Charpy Test Results - NIST Contribution to an International Study (National Institute of Standards and Technology, Boulder, CO), NIST Technical Note (TN) 2089. 10.6028/jres.125.015 PMC9097812 35573858

